# Hospital Notes—Sarcoma of Left Wrist

**Published:** 1880-05

**Authors:** Frederick Peterson, C. C. F. Gay

**Affiliations:** House Physician at the Buffalo General Hospital


### HOSPITAL NOTES.

REPORTED BY DR. FREDERICK PETERSON, HOUSE PHYSICIAN AT THE BUFFALO GENERAL HOSPITAL.—SERVICE OF DR. C. C. F. GAY.

### Sarcoma of Left Wrist.

Nelson Sine, entered Hospital, April 15, 1879; single; age 28; American; brakesman; joint of left wrist swollen, and discharges from back a thin brownish fluid; sprained the joint five months before; flax-seed cataplasms were applied.

April 19. Dr. Gay cut down upon back of carpus, through a cineritious and suspicious-looking tissue; operation under ether; carpal bones found unusually soft; bandaged with sponge in wound as compress.

April 21. Immersed hand in water at temperature of 100°, until

May 3d. Hot fomentations ordered.

May 10. An abscess has formed in front of carpus; opened it to his relief.

May 29. Found it necessary to amputate fore-arm three inches above wrist. That the operation was required, a number of other members of the staff believed. Specimens from the carpus and sections even of portions of the healthy-appearing flaps showed the presence of the round cells of this form of sarcoma under the microscope. Ordered Fowler's Solution gtt. iij. three times daily. Stump healed remarkably quick, and patient grew ruddy and full-faced under generous diet.

June 23. Discharged in perfect physical health; stump a very handsome one. It is said, however, that quick healing is a characteristic of sarcoma, and that it is sure to return.

